# Functional Analysis of KIF3A and KIF3B during Spermiogenesis of Chinese Mitten Crab *Eriocheir sinensis*

**DOI:** 10.1371/journal.pone.0097645

**Published:** 2014-05-28

**Authors:** Yang Lu, Qi Wang, Da-Hui Wang, Hong Zhou, Yan-Jun Hu, Wan-Xi Yang

**Affiliations:** 1 The Sperm Laboratory, College of Life Sciences, Zhejiang University, Hangzhou, China; 2 Department of Reproductive Endocrinology, Women's Hospital, School of Medicine, Zhejiang University, Hangzhou, China; St. Georges University of London, United Kingdom

## Abstract

**Background:**

Spermatogenesis represents the transformation process at the level of cellular development. KIF3A and KIF3B are believed to play some roles in the assembly and maintenance of flagella, intracellular transport of materials including organelles and proteins, and other unknown functions during this process. During spermatogenesis in *Eriocheir sinensis*, if the sperm shaping machinery is dependent on KIF3A and KIF3B remains unknown.

**Methodology/Principal Findings:**

The cDNA of KIF3A and KIF3B were obtained by designing degenerate primers, 3′RACE, and 5′RACE. We detected the genetic presence of *kif3a* and *kif3b* in the heart, muscle, liver, gill, and testis of *E. sinensis* through RT-PCR. By western blot analysis, the protein presence of KIF3A and KIF3B in heart, muscle, gill, and testis reflected the content in protein level. Using *in situ* hybridization and immunofluorescence, we could track the dynamic location of KIF3A and KIF3B during different developmental phases of sperm. KIF3A and KIF3B were found surrounding the nucleus in early spermatids. In intermediate spermatids, these proteins expressed at high levels around the nucleus and extended to the final phase. During the nuclear shaping period, KIF3A and KIF3B reached their maximum in the late spermatids and were located around the nucleus and concentrated in the acrosome to some extent.

**Conclusions/Significance:**

Our results revealed that KIF3A and KIF3B were involved in the nuclear and cellular morphogenesis at the levels of mRNA and protein. These proteins can potentially facilitate the intracellular transport of organelles, proteins, and other cargoes. The results represent the functions of KIF3A and KIF3B in the spermatogenesis of Crustacea and clarify phylogenetic relationships among the Decapoda.

## Introduction

Spermiogenesis represents one of the most complicated morphological transformation procedures and is divided into three main phases along with proliferation and differentiation from diploid spermatogonia to haploid spermatozoa [1], [6], [12], [14], [17]. Firstly, mitosis induces the spermatogonia to develop into two identical primary spermatocytes. Then through meiosis each primary spermatocyte develops into the secondary spermatocyte [11], [12], [16]. Subsequently, the spermatids develop into mature spermatozoa with nucleus elongation and condensation, the formation of a mid-piece, the remodeling of the acrosome, and the reorganization of the intracellular organelle [1], [8], [14]. But the mature sperm of various species may differ from each other. *Eriocheir sinensis*, also called Chinese mitten crab, is a species of the class Crustacea, Decapoda, Brachyura [1], [2]. Compared to other species, the spermatozoon of *E. sinensis* is of very peculiar shape. The nucleus of the spermatozoon looks just like a cap that surrounds the oval acrosome [1], [7]. The acrosome consists of the acrosomal tubule, the apical cap (AC), and the acrosomal vesicle [7]. The mature spermatozoon has no flagellar tail that is mainly responsible for the movement of the spermatozoa of other species. However, there are about 20 radial arms extruding from the outside of the cup-shaped nucleus. Whether the content of radial arms belongs to the microfilament or the microtubule is still a matter of debate. *E. sinensis* is a commercially important seafood crab due to its delicious taste and rich nutrition. Studies of spermatogenesis in *E. sinensis* are essential for the maintenance and improvement of reproduction in general. *E. sinensis* has been used as a suitable model for general studies of spermatogenesis [1], [7]. The morphological variations of the nucleus during spermatogenesis are regarded as evidences for analyzing evolution of Decapoda, and the phylogenetic status of this species can be inferred as well [10], [11]. However, the specific molecular functions of the motor proteins are as yet less known than the morphology [9]. An example is given by the gap junctions between cells that improve spermatogenesis, but the molecular mechanisms are still far from being fully understood [9].

Most striking morphological transformations occur with the microtubules (MTs), microfilaments, and the motor proteins associating with them. The transport and sorting of cargoes in tail and manchette, the specific structure surrounding the nucleus of the spermatid, are all dependent on the kinesin superfamily of proteins (KIFs) [13], [17]. KIFs can make use of ATP hydrolysis to produce energy for transporting a series of organelles, protein complexes, and vesicles [18]. Kinesin-2 motors are mainly heterotrimeric proteins consisting of two different motor subunits and one accessory subunit [19]. The motor subunit is composed of the N-terminal domain, a rod domain, and the C-terminal globular domain [17], [20], [21]. KIF3A can assemble with KIF3B or KIF3C, while KIF3B can not assemble with KIF3C [22]. The heterodimer and the accessory subunit, KAP3, can combine as a heterotrimeric motor protein, KIF3. KAP3 has the location to associate with cargoes through small G proteins, whereas KIF3A/KIF3B bond to the MTs [23]. The homolog of KIF3 in sea urchin has been reported to be a heterotrimer composed of SpKRP85, SpKRP95 and SpKAP115 [24]–[26]. KIF3 is responsible for the formation and elongation of cilia along with the central pair of MTs [19]. OSM-3 functions in the anterograde transport of cargoes that can mediate sensory ciliary growth in sensory neurons and inner labial neurons [27]. It is speculated that KIF3B promotes the aggregation of mitochondria with the formation of IFT and IMT [28]. There is evidence showing that the link between kinesin-2 and IFT depends on IFT20 and KIF3B. IFT20 connects directly with IFT57 of the IFT complex consisting of IFT57, IFT88, and IFT52 [29]. Mice with *kif3a* knockdown are lethal to embryos before any differential organogenesis takes place [30], [31]. The abnormal primary cilia caused by *kif3a* deficiency in mice develop to polycystic kidney disease (PKD), the rapid formation of kidney cysts, and false planar cell polarity [30], [31]. KIF3A associates with β-catenin to mediate the epithelia from some reports. NEK1 is another protein related to the formation of kidney cysts and PKD. It is the first time to report that KIF3A is the interacting protein partner of NEK1 during the genesis of PKD [32], [33]. KIF3A, therefore, links the development of cilia to regulate the cell cycle. The three signaling cascades related to cilia involve the following pathways: Shh pathway, PDGFR pathway and Wnt pathway. These are also mediated by KIF3 in the mediation of tumorigenesis [34].

In mammals, crustaceans, and cephalopods, KIFs are associated with the reorganization of nucleus and acrosome during spermatogenesis [3], [4], [5]. KRP85/KRP95 localize in the flagella and the midpiece of the sperm in sea urchin and sand dollar [35]. In the midpiece of the sperm, the localization of the centrosome is the same as the localization of KRP85/KRP95. From the observation on the location of the mRNA signal, KIF3A/KIF3B are responsible for IMT, IFT, the communication of cells, and the elongation of the nucleus [36], [37]. RNF33 can interact with KIF3 independent of KAP3. As RNF33 is mediated by a signaling pathway named TNFα-NFκb pathway which is involved in sex differentiation and reproduction, RNF33-KIF3 interaction may be indispensible during spermiogenesis [28], [38]. KIFC1 belongs to the kinesin-14 superfamily and is expressed in the acrosome complex and subacrosomal space in *E. sinensis* [2], [14], [15]. The manchette is also shown to be associated with KIFC1 in the remodeling of the nucleus and cytoplasm [16]. Whether KIF3A/KIF3B play a similar function during spermatogenesis in *E. sinensis* deserves further investigation. Some reports have demonstrated that KIF3A and KIF3B are responsible for IFT, the maintenance and elongation of a flagellar tail in other species, while in *E. sinensis* this needs still further research on [3], [4], [5]. We hypothesize that KIF3A and KIF3B may participate in the transport of vesicles and other cargoes; they may also be responsible for acrosome biogenesis and nuclear reshaping. We speculate that KIF3A and KIF3B may have a tight connection and a similar distribution during spermatogenesis in *E. sinensis*.

## Materials and Methods

### Animals and Sampling

Specimens of *E. sinensis* were purchased from Hangzhou Luo Jia Zhuang Farmer's Market from September, 2012 to December, 2013. Thirty adult male individuals were selected and transported to the Sperm Laboratory at Zhejiang University in sea water tanks with aeration facilities. Following temporary maintenance, crabs were anesthetized on ice and dissected to obtain the hepatopancreas, heart, muscle, gill, and testis. These samples were quickly frozen in liquid nitrogen and used for RNA and protein extraction. Testes and seminal vesicles from ten crabs were fixed with 4% PFA-PBS (ph 7.4) for *in situ* hybridization. Meanwhile, five other different individuals were subjected to fixation overnight in 4% paraformaldehyde-phosphate buffered saline (PBS) (pH 7.4) at 4°C for Immunofluorescence (IF) studies.

No approval for experimentation on Chinese mitten crab *E. sinensis* is needed in China.

### RNA extraction and reverse transcription

Total RNA from the testis, heart, muscle, gill, and hepatopancreas of *E. sinensis* was extracted using Phase Lock Gel™ Heavy with Trizol A^+^ reagent (Tiangen Biotech, Beijing, China). These samples were treated with chloroform, isopropanol, and 75% alcohol to get RNA. Then RNA was stored at −80°C for subsequent experiments. Reverse transcription was carried out by using PrimeScriptH RT reagent Kit (Takara, Dalian, China). 5′ RACE and 3′ RACE reverse transcription assays use Smart RACE cDNA Amplification Kit (CloneTech, Mountain View, USA). Their products were stored at −20°C for subsequent assays.

### Rapid-amplification of cDNA ends (RACE)

Degenerate primers were designed by using Primer Premier 5.0 based on the conservative property of protein sequences in other species. Gene specific primers of *kif3a* and *kif3b* were designed by Oligo 6 and Primer Premier 5.0. All the primers of *kif3a* and *kif3b* were synthesized by Shanghai Sangon Biological Engineering Technology Company. The primers for cloning *kif3a* and *kif3b* were shown in Table 1 and Table 2. The Touchdown PCR (TD-PCR) strategy for obtaining intermediate fragments is shown as follows: 94°C for 5 min, 14 cycles for a touch down program (94°C for 30 s, 55°C for 30 s and 72°C for 30 s, followed by 0.5°C decreasing per cycle), then 31 cycles for another program (30 s at 94°C, 30 s at 48°C and 30 s at 72°C), and 72°C for 10 min. Then AxyPrep DNA Gel Extraction Kit (Axygen, Silicon Valley, USA) was used to extract the target fragments. These fragments were inserted into PMD18-T (Takara, Dalian, China) for ligation. The ligation product was transformed into *Escherichia coli* DH5α for blue and white screening. The positive recombinant clone was sent to BioSune Company, Shanghai, China for sequencing.

**Table 1:** The primers of *kif3a* used in this study.

Primer name	Sequence	Purpose
KIF3A-F1	ATCTTCGCTTAYGGNCARACNGG	PCR
KIF3A-F2	AACCTGGTGGAYYTNGCNGG	PCR
KIF3A-F3	GGAAATATCMGGGTNTTYTGYMG	PCR
KIF3A-F4	TCGGGGGAAACTCCAAGAC	PCR
KIF3A-F5	ACGAGACCATCAGCACCCG	PCR
KIF3A-F6	CGTGAAGGTGGTGGTG	PCR
KIF3A-R1	GTCARCTTAGAGTTNCKRTANGG	PCR
KIF3A-R2	CCWGTYTGTCCRTANGCRAA	PCR
KIF3A-R3	GCTGCCATTCTCCAATATCTTCRTTCCARTG	PCR
KIF3A-R4	TTCAGCTGCCATTCTCCAATRTCYTCRTTC	PCR
KIF3A-R5	CTTTGTCTTTCTGAACCTGC	PCR
KIF3A-R6	CAGCCTCTGTCCTGTTGC	PCR
3′-KIF3A-F1	CTGGACACTCCTTATGGCGGCTA	3′RACE
3′-KIF3A-F2	ACCAGGAGATGATAGAGCGATAC	3′RACE
5′-KIF3A-R1	AAGATGTGTGCGAAGGAGTTTGGAATGA	5′RACE
5′-KIF3A-R2	CCGCTCAGCACACTCAATGGTGATGGT	5′RACE
β-actin F	GCATCCACGAGACCACTTACA	Positive control
β-actin R	CTCCTGCTTGCTGATCCACATC	Positive control
KIF3A-S-F	AACCTTGCTGCTCGTCCCATT	RT-PCR and ISH
KIF3A-S-R	CCCCGACCGCTCTGTTCTTAT	RT-PCR and ISH
KIF3A-P-F	CGCGGATCCCCGTGGTGCCAGCCCGTAA	Prokaryotic expression
KIF3A-P-R	CCGGAATTCTCCATCTCCCCAGCATTGTG	Prokaryotic expression

**Table 2:** The primers of *kif3b* used in this study.

Primer name	Sequence	Purpose
KIF3B-F1	GACCGTCATGGTGGCNAAYATHGG	PCR
KIF3B-R1	CTRGTRTGNTTGCTCGTCGTCTCCC	PCR
3′-KIF3B-F1	AAGAGCACTCACATCCCTTACAGA	3′RACE
5′-KIF3B-R1	CCTCGTTCACCTTGGGCTTGTTCTTGATGT	5′RACE
5′-KIF3B-R2	TGTGCTCAAACGAGTTGGGAATGACG	5′RACE
β-actin F	GCATCCACGAGACCACTTACA	Positive control
β-actin R	CTCCTGCTTGCTGATCCACATC	Positive control
KIF3B-S-F	GGGACCATTTTTGCGTAT	RT-PCR and ISH
KIF3B-S-R	TGTGACCGGGAGCTGTGT	RT-PCR and ISH

The specific primers (GSPs) for 3′RACE and 5′RACE were designed by using Primer Premier 5.0 (Table 1 and Table 2). Several other primers in the RACE kits were used with GSPs. The 3′RACE protocol was as follows: 94°C for 5 min; 6 cycles of 94°C for 30 s, 55°C (decreased by 0.5°C /cycle) for 30 s, and 72°C for 45 s, then 33 cycles of 94°C for 30 s, 52°C for 30 s, and 72°C for 45 s, 72°C for 10 min for extension. The 5′RACE program is shown as follows: 94°C for 5 min; 10 cycles of 94°C for 30 s, 62°C (decreased by 0.5°C/cycle) for 30 s, and 72°C for 45 s, then 33 cycles of 94°C for 30 s, 57°C for 30 s, and 72°C for 45 s, 72°C for 10 min for extension. Then the products were dealt with as above in order to obtain the full sequences.

### Sequence analysis and phylogenetic analysis

BLAST service provided by the National Center for Biotechnology Information (NCBI) (http://www.ncbi.nlm.nih.gov/) was used for unknown sequence analysis. The cDNA and protein sequence were analyzed and aligned by the Vector NT110 (Invitrogen, California, USA) software to obtain the conserved sequences. The phylogenetic trees of KIF3A and KIF3B were constructed through comparison with the homologues in other species using the software Mega5. Genebank accession numbers of KIF3A proteins in protein alignment are as follows: *Bos taurus* (NP_001193783.1), *Culex quinquefasciatus* (EDS31781.1), *Cynops orientalis* (ADM26621.1), *Danio rerio* (AAH77150.1), *Eriocheir sinensis* (JN645277), *Gallus gallus* (NP_001025793.1), *Glyptapanteles indiensis* (ACE75374.1), *Homo sapiens* (AAH20890.1), *Loa loa* (EFO18808.1), *Oncorhynchus mykiss* (NP_001158607.1), *Pan troglodytes* (NP_001233450.1). Genebank accession numbers of KIF3B proteins in protein alignment are also as follows: *Aureococcus anophagefferens* (EGB05251.1), *Columba livia* (XP_005499585.1), *Danio rerio* (NP_001093615.1), *Drosophila melanogaster* (AGB94420.1), *Eriocheir sinensis* (KF751391), *Gallus gallus* (NP_001012852.1), *Mus musculus* (NP_032470.3), *Octopus tankahkeei* (AEL16465.1), *Xenopus laevis* (NP_001081489.1). The analysis of the secondary structure and the 3-D structure were processed by PROSITE (http://prosite.expasy.org/) and I-TASSER (http://zhanglab.ccmb.med.umich.edu/I-TASSER) [39], [40].

### Analysis of tissue distribution of *kif3a* and *kif3b* mRNA

The reverse transcription of mRNA in different tissues in *E. sinensis* was used to provide the cDNA for Semi-quantitative RT-PCR analysis of *kif3a* and *kif3b* expression in different tissues. The PrimeScript RT reagent Kit (Takara, Dalian, China) was needed for the analysis. The two pairs of specific primers (Table 1 and Table 2) were designed by Primer Premier 5 software. The primers of β-actin (Table 1 and Table 2) were designed for the positive control. The PCR assay is shown as follows: 94°C for 5 min; 35 cycles of 94°C for 30 s, 55°C for 30 s, and 72°C for 30 s; 72°C for 10 min for the final extension. The final PCR products were detected by agar gel electrophoresis. The expression quantities of *kif3a* and *kif3b* were analyzed through the Quantity 1 (version 4.4.0) software from Tanon Science &Technology Co., Ltd.

### *In situ* hybridization (ISH)

#### Tissue preparation

Testes and seminal vesicles were embedded into the optimum cutting temperature (O.C.T) compound (VWR Corporate, Radnor, PA, USA). Then these were sectioned at about 10 µm thickness with a freezing microtome. The samples were kept at −20°C and an RNase-free atmosphere for the whole process. Then the samples were rapidly stored at −80°C for the following experiments.

#### Riboprobe synthesis

The Riboprobes were obtained by designing specific primers for ISH (Table 1 and Table 2). Gene-specific primers for ISH were designed by Oligo 6 and Primer Premier 5.0. All the primers for ISH were synthesized by Shanghai Sangon Biological Engineering Technology Company. The fragment of *kif3a* for ISH is 397 bp and the fragment of *kif3b* for ISH is 380 bp. The PCR program was as follows: 94°C for 5 min; 35 cycles of 94°C for 30 s, 55°C for 30 s, and 72°C for 30 s; 72°C for 10 min for the final extension. The fragments of *kif3a* and *kif3b* were all inserted into a PGEM-T EASY Vector (Promega, Beijing, China) for ligation. Then the products of ligation were transformed into competent cells ((*Escherichia coli* DH5α) for blue and white screening. The positive recombinant clone was sent to BioSune Company, Shanghai, China for sequencing. The two fragments were linearized and transcribed with a T7 promoter *in vitro*. Ethanol and LiCl were used to precipitate the riboprobes. Then the products were submerged in DEPC-treated H_2_O. The spectrophotometer and nucleic acid electrophoresis were used to assess the concentration and quality of riboprobes.

#### Prehybridization and hybridization

The sections were placed at room temperature in RNase-free atmosphere for about 10 min. The sections were fixed with 4% paraformaldehyde (PFA, pH 7.4) for about 10 min. The 0.1% diethylpyrocarbonate (DEPC)-activated 0.1M phosphate-buffered saline (PBS, pH 7.4) was used for rinsing the sections twice. Then they were equilibrated for 15 min in 5×SSC (sodium chloride 0.75 M, sodium citrate 0.075 M, pH 7.0). The prehybridization was composed of 50% deionized formamide, 40 µg/mL denatured salmon sperm DNA, and 5×SSC solution. Then the sections were placed in prehybridization solution for 2 h at 55°C to 58°C. Approximately 300 ng/ml of denatured and digoxigenin (DIG)-labeled riboprobes were added to the prehybridization solution to obtain the hybridization buffer. The testis sections were placed in the hybridization buffer overnight at 57°C.After that, the sections were rinsed for 30 min in 2×SSC at room temperature, then 1 h in 2×SSC at 65°C, and 1 h in 0.1×SSC at 65°C.

### Detection of the signal

Buffer I (0.1 M Tris-hydrochloride, 0.15 M NaCl, pH 7.5) was used for equilibrating the sections for 5 min. Then the sections were incubated in DIG Buffer I, consisting of 1∶2000 anti-DIG alkaline phosphatase conjugated Fab fragments (Roche, Branford, USA) and 0.5% (w/v) blocking reagent (Roche, Branford, USA) for about 2 h at room temperature. Then the sections were washed three times with Buffer I for 15 min each time and then equilibrated in Buffer II (0.1 M Tris-HCl, 0.1 M NaCl, 0.05 M MgCl2, pH 9.5) for 15 min at room temperature. The chromogenic agent (330 µg/mL nitroblue tetrazolium chloride and (NBT) and 165 µg/mL 5-bromo-4-chloro-3-indolyl-phosphate (BCIP) in Buffer II) (Promega, Beijing, China) were used to color the sections in the dark for 2 h at room temperature. The final reaction ceased through rinsing the sections in 1×TE buffer (10 mM Tris-HCl, 1 mM EDTA, pH 8.0). The nonspecific stain was removed in 95% ethanol for 1.5 h. Deionized water was used to wash the precipitated Tris crystals for 15 min. A series of ethanol at 50, 75, 95, 100% level were used to dehydrate the sections. The sections were infiltrated in xylol and mounted. A Nikon Eclipse E80i microscope (Nikon, Tokyo, Japan) was used to observe the signals.

### Western Blot and Immunofluorescence (IF)

#### Prokaryotic expression

The sequence of *kif3a* was obtained with the primers (Table 1) containing a BamH I restriction site on the 5′ end and an EcoR I restriction site on the 3′ end. The plasmid of *kif3a* was digested and ligated into pET28a (Invitrogen Life Technologies, California, USA) to develop the recombined plasmid. Then this was transformed into BL21 and subsequently the colony was inoculated into 200 ml Luria-Bertani medium with 50 ml kanamycin (100 mg/ml). Then this was shaken with 200 rpm at 37°C to reach 0.8 in OD_600_. IPTG (Isopropyl-b-D-thiogalactoside) was added into the medium to reach a concentration of 1 Mm. It was shaken with 200 rpm at 37°C for 12 h and then dealt with ultrasonication. The supernatant was purified by nickel-nitrilotriacetic acid agarose affinity chromatography according to the QIA expressionist manual (Qiagen, Frankfurt, Germany) to obtain the HIS-KIF3A protein. Then the protein was used to develop antibody in rabbit. The purified protein mixed with Freund's complete adjuvant was injected into the rabbit. The next two injections were scheduled after 2days and 27days. After 34 days, we extracted the blood from the rabbit and purified the blood for extracting the serum. The serum contained rabbit anti-KIF3A antibody. The rabbit anti-β-actin (BIOS, Shanghai, China), FITC conjugated monoclonal anti-tubulin (Sigma, St. Louis, Mo., USA), HRP conjugated goat anti-rabbit IgG (Immunology consultants Laboratory, Inc), and Texas Red conjugated affinipure goat anti-rabbit IgG (Protein Tech Group, Inc) were prepared for the next experiments.

#### Western blot

Several tissues from *E. sinensis* were placed into RIPA Lysis Buffer (Solarbio, Shanghai, China) with protease inhibitors for homogenizing the tissues. The supernatants mixed with 5×SDS sample buffer were used to load and proteins were separated on 12% gels (SDS-PAGE). The proteins were transferred on the surface of the PVDF membrane (Bio-Rad, California, USA). The membrane was blocked in 4% BSA in PBST (PBS with 0.5% Tween 20) for 1 h and then incubated with rabbit anti-KIF3A antibody (diluted 1∶250) in 4% BSA. Then PBST was used three times to wash for 15 min each time. The PVDF membrane was then incubated for 1 h in secondary antibody HRP-conjugated goat anti-rabbit IgG (diluted 1∶800) in 4% BSA. Then the PVDF membrane was washed three times for 15 min each time. SuperSignal West Pico Trial Kit (Thermo, Massachusetts, USA) was used for band detection with a chemiluminescence imaging device (Chemiscope 3400) (Shanghai, China).

#### Immunofluorescence (IF)

Testes and seminal vesicles were taken out and fixed in 4% paraformaldehyde (PFA, pH 7.4) overnight. The PBS was used for rinsing the samples three times for 15 min each time. Then they were incubated in PBS with 0.5 M sucrose overnight. Then the samples were embedded in optimum cutting temperature (O.C.T) compound for sectioning. The sections were washed in PBST (PBS with 0.3% Triton X-100) three times for 15 min each time. Then the sections were blocked in 5% BSA dissolved in PBST (0.1% Triton X-100) for 1 h. After that, the sections were incubated in 5% BSA dissolved in PBST (0.1% Triton X-100) with anti-KIF3A (diluted 1∶200) for 2 h at 37°C. Then the sections were rinsed three times for 15 min each in PBST (0.1% Triton X-100). The sections were incubated in 5% BSA dissolved in PBST (0.1% Triton X-100) with Texas Red goat anti-rabbit IgG (diluted 1∶200) or FITC-anti-Tubulin conjugated goat anti-mouse IgG (Sigma, St. Louis, MO, USA) for 1 h at 37°C. DAPI (Beyotime, Dalian, China) was used for staining the nuclei for 5 min. The sections were observed with a Nikon Eclipse E80i microscope (Nikon, Tokyo, Japan) and Nikon Eclipse E80i microscope (Nikon, Tokyo, Japan).

## Results

### *Kif3a* and *kif3b* sequence analysis

**Figure 1:**
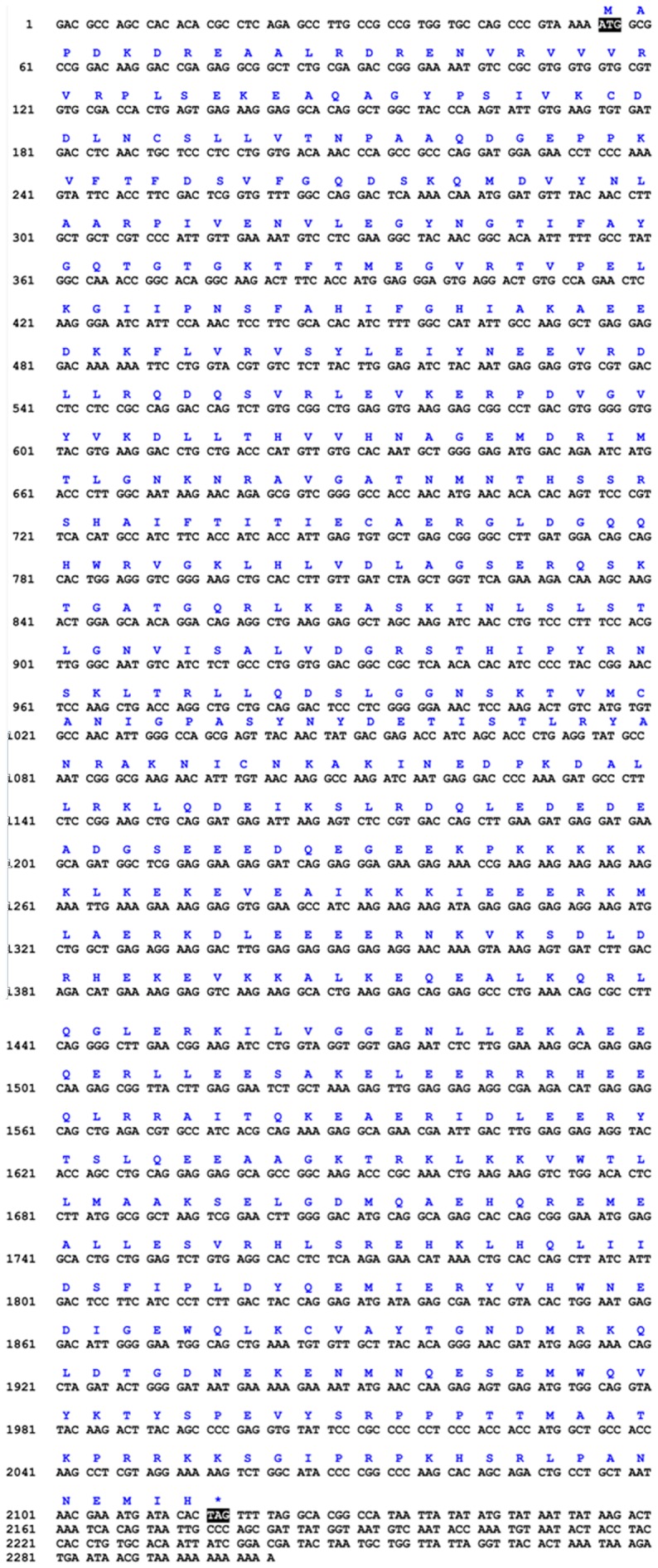
Full-length cDNA of the *kif3a* in *E. sinensis*. The amino acid sequence is deduced from the nucleotide sequence. This figure shows that the full-length cDNA of *kif3a* consists of a 54 bp 5′ untranslated region, a 187 bp 3′ untranslated region and a 2061 bp open reading frame. The open reading frame encodes 687 amino acids.

**Figure 2:**
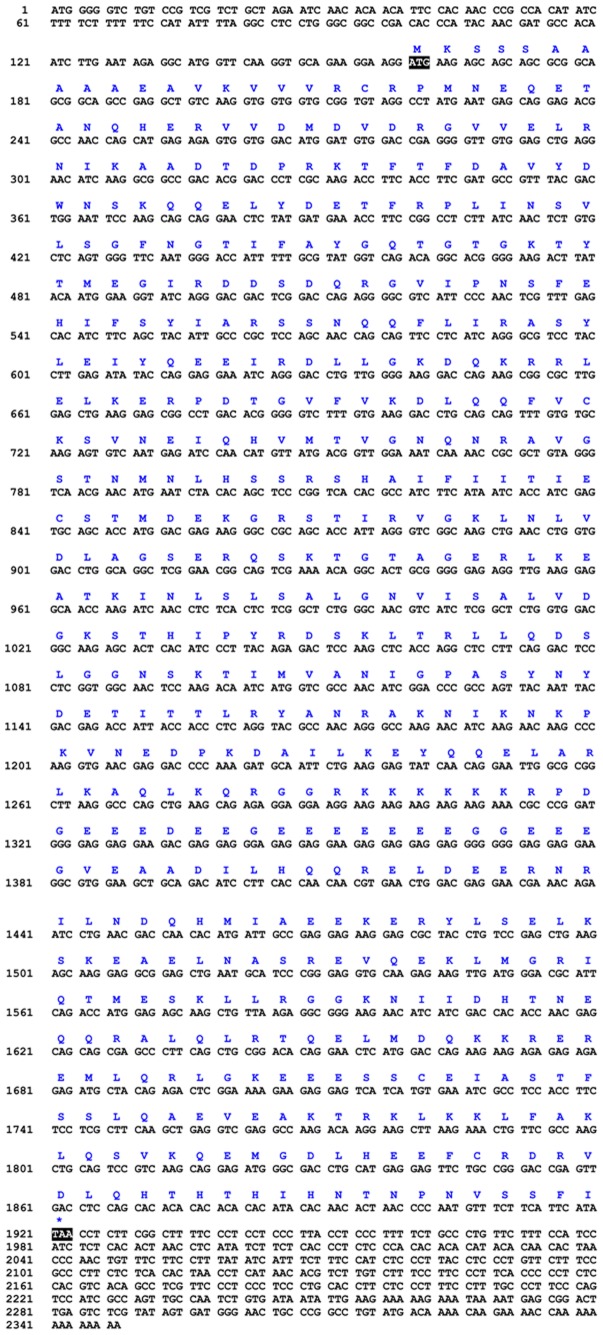
Full-length cDNA of the *kif3b* in *E. sinensis*. The amino acid sequence can be deduced from the nucleotide sequence. This figure shows that the full-length cDNA of *kif3b* consists of a 159 bp 5′untranslated region, a 428 bp 3′ untranslated region and a 1761 bp open reading frame. The open reading frame encodes 587 amino acids.

We used degenerate primers to obtain intermediate fragments of *kif3a* and *kif3b*. Subsequently, we used specific primers to amplify the rest of the sequences through 3′ RACE and 5′RACE. The full-length cDNA of *kif3a* consisted of a 54 bp 5′ untranslated region, a 187 bp 3′ untranslated region and a 2061 bp open reading frame. The open reading frame encoded 687 amino acids (Fig. 1). However, the full-length cDNA of *kif3b* consisted of a 159 bp 5′ untranslated region, a 428 bp 3′ untranslated region and a 1761 bp open reading frame. The open reading frame encoded 587 amino acids (Fig. 2).

### The alignment of KIF3A and KIF3B protein sequence and resulting phylogenetic tree

**Figure 3:**
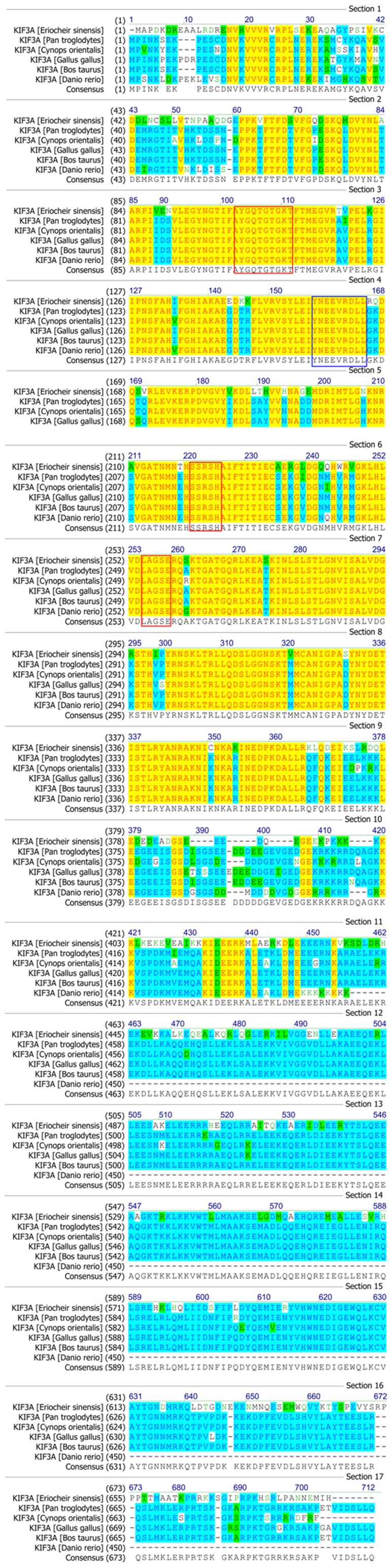
Comparison of the KIF3A protein in *E. sinensis* with homologues of other species. This figure shows the amino acid alignment of KIF3A with its homologues using Vector NTI10 (Invitrogen, California, USA). The AYGXTGXGKX, SSRSH, and LAGSE sequences (red frame) are the putative ATP-binding domain, while the YXXXXXDLL sequence (blue frame) is the putative microtubule-binding motif. KIF3A in *E. sinensis* shows a 64.1, 64.1, 64.5, 64.1, and 44.2% identity with the homologues in *Pan troglodytes*, *Cynops orientalis*, *Gallus gallus*, *Bos taurus*, and *Danio rerio*, respectively.

**Figure 4:**
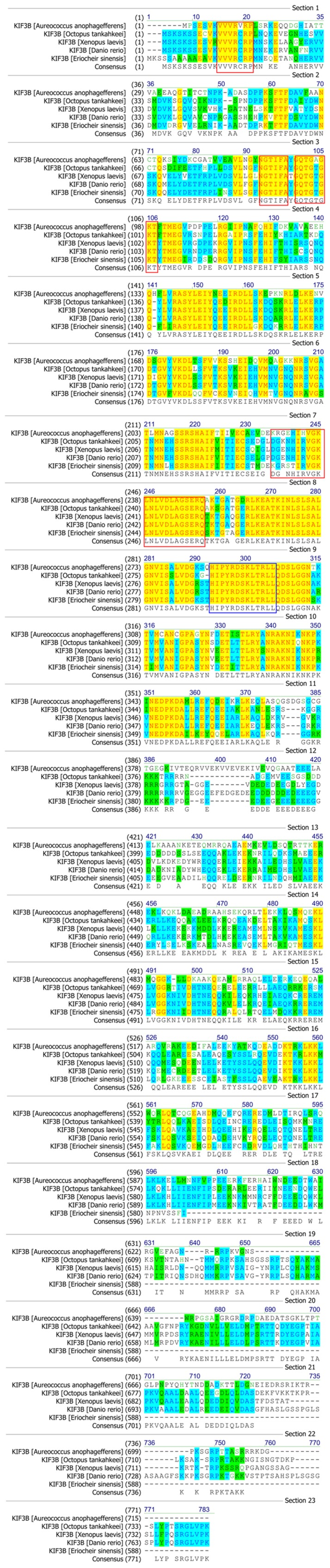
Comparison of the KIF3B protein in *E. sinensis* with homologues of other species. This figure shows the amino acid alignment of KIF3B with its homologues using Vector NTI10 (Invitrogen, California, USA). The VVVRCRP, NGTIFA, GQTGTGKT, and DGENHIRVGKLNLVDLAGSERQ sequences (red frame) are the putative ATP-binding domain, while the HIPYRDSKLTRLL sequence (blue frame) is the putative microtubule-binding motif. KIF3B in *E. sinensis* shows a 40.3, 47.1, 49.5 and 48.6% identity with the homologues in *Aureococcus anophagefferens*, *Octopus tankahkeei*, *Xenopus laevis*, and *Danio rerio*, respectively. The identity between KIF3A and KIF3B is about 41.6% in *E. sinensis*.

**Figure 5:**
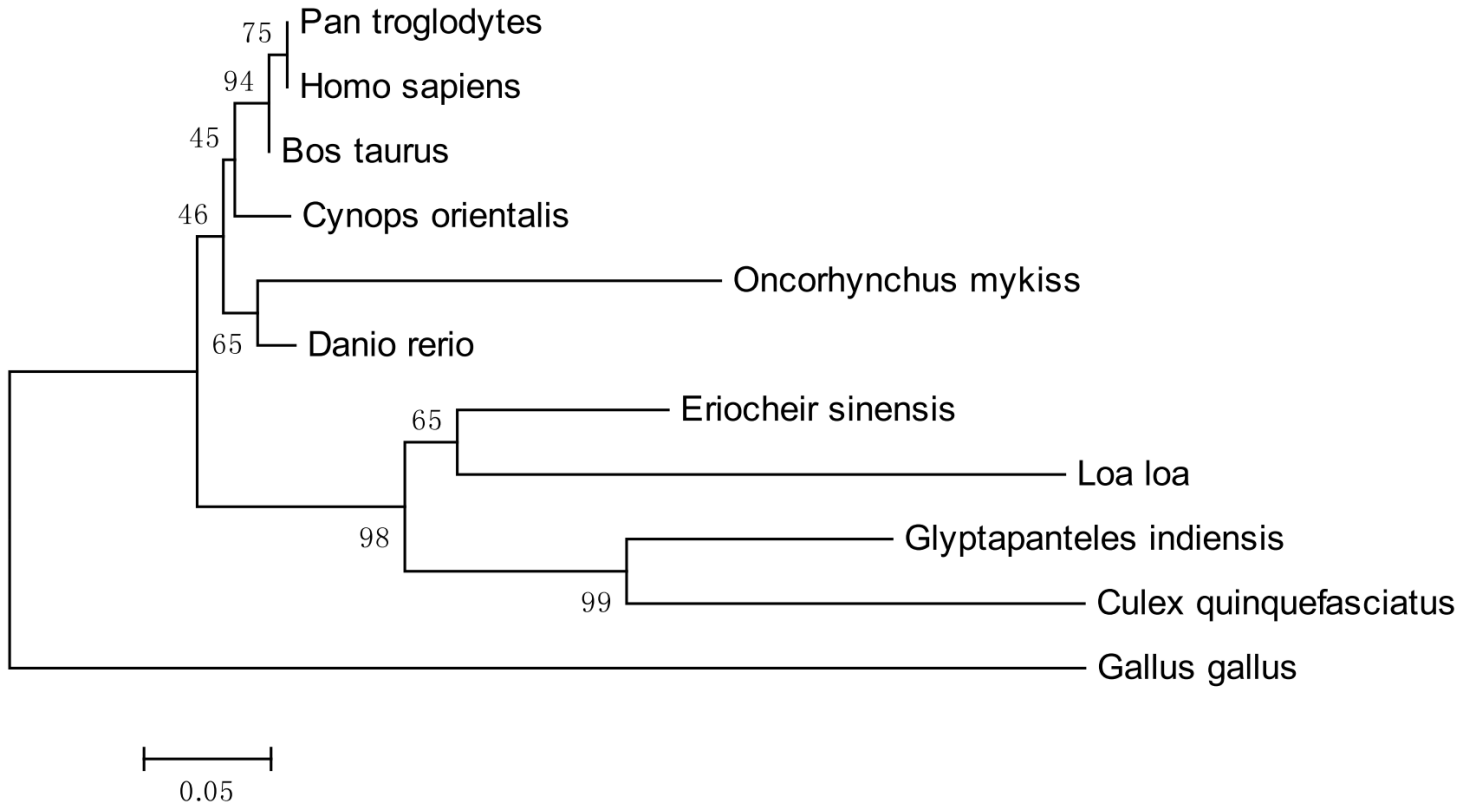
The phylogenetic tree of KIF3A protein and its homologues. This figure shows the phylogenetic tree of KIF3A and its homologues in other species that were constructed through the neighbor-joining method in Mega 5 (version 5.0) software. We examined the KIF3A from *E. sinensis, Oncorhynchus mykiss, Bos taurus, Cynops orientalis, Danio rerio, Pan troglodytes, Homo sapiens, Gallus gallus, Glytapanteles indiensis, Culex quinquefasciatus, and Loa loa*. The putative protein of *E. sinensis* is most closely related to *Loa loa*.

**Figure 6:**
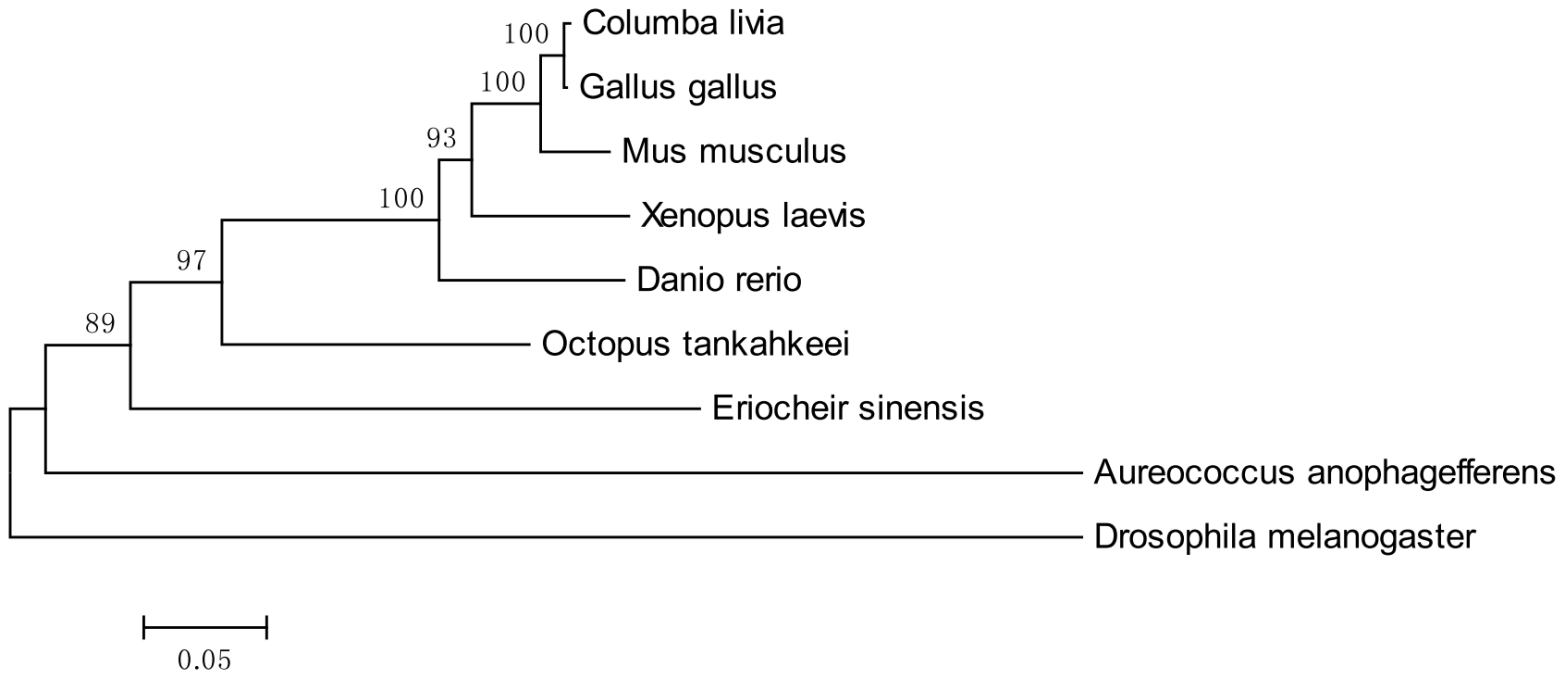
The phylogenetic tree of KIF3B protein and its homologues. This figure shows the phylogenetic tree of KIF3B and its homologues in other species that were constructed through the neighbor-joining method in Mega 5 (version 5.0) software. We examined the KIF3B from *E. sinensis*, *Mus musculus*, *Danio rerio*, *Aureococcus anophagefferens*, *Columba livia*, *Drosophila melanogaster*, *Gallus gallus*, *Octopus tankahkeei*, and *Xenopus laevis*. The putative protein of *E. sinensis* is most closely related to *Octopus tankahkeei*.

The colored blocks (Fig. 3 and Fig. 4) represent different conservative levels of amino acids. The yellow blocks indicate identical amino acids and the sky blue blocks indicate conserved amino acids. The green blocks indicate weakly similar amino acids, while the others represent non-similar amino acids. The AYGXTGXGKX, SSRSH, and LAGSE sequences (red frame) are the putative ATP-binding domain, while the YXXXXXDLL sequence (blue frame) represents the putative microtubule-binding motif of KIF3A [15], [32], [35], [41]. The sequenced KIF3A shows a 64.1, 64.1, 64.5, 64.1, and 44.2% identity with the homologues in *Pan troglodytes*, *Cynops orientalis*, *Gallus gallus*, *Bos taurus*, and *Danio rerio*, respectively (Fig. 3). The VVVRCRP, NGTIFA, GQTGTGKT, and DGENHIRVGKLNLVDLAGSERQ sequences (red frame) are the putative ATP-binding domains, while the HIPYRDSKLTRLL sequence (blue frame) represents the putative microtubule-binding motif of KIF3B. The sequenced KIF3B shows a 40.3, 47.1, 49.5 and 48.6% identity with the homologues in *Aureococcus anophagefferens*, *Octopus tankahkeei*, *Xenopus laevis*, and *Danio rerio*, respectively. The identity between KIF3A and KIF3B is about 41.6% in *E. sinensis* (Fig. 4). The phylogenetic analysis of KIF3A and KIF3B demonstrates that the putative KIF3A protein of *E. sinensis* is most closely related to *Loa loa* (Fig. 5), while the putative KIF3B protein of *E. sinensis* is most closely related to *Octopus tankahkeei* (Fig. 6).

### Structural analysis of KIF3A and KIF3B protein

**Figure 7:**
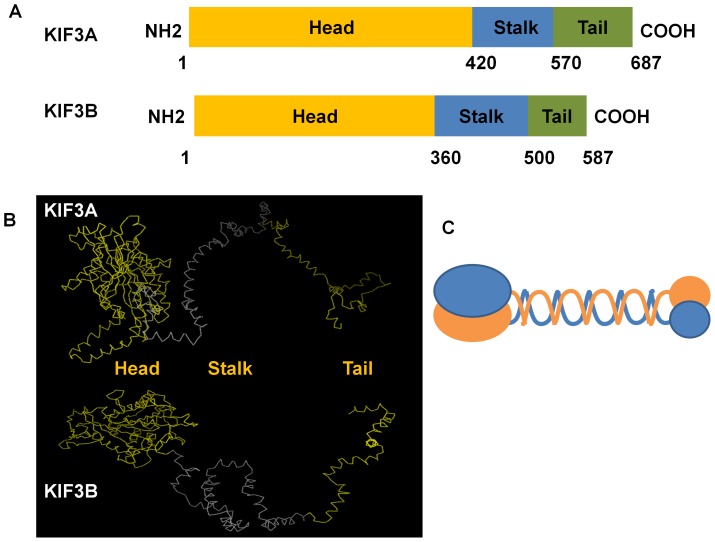
The major structural features of KIF3A and KIF3B in *E. sinensis*. This figure shows the three structural domains in KIF3A and KIF3B. They all have three domains consisting of the head domain, the stalk domain, and the tail domain. As for KIF3A, the N-terminal (1–420 aa) contains the conserved head (yellow bar) that can move along the microtubules, the stalk domain (421–570 aa) can form an extended coiled-coil region (blue bar), and the C-terminal (571–687 aa) may contain a divergent tail (green bar) that carries a series of cargoes. As for KIF3B, the N-terminal (1–360 aa) contains the conserved head (yellow bar) that can move along the microtubules, the stalk domain (361–500 aa) can form an extended coiled-coil region (blue bar), and the C-terminal (501–587 aa) contains divergent tail (green bar) that carries a series of cargoes. (B) The figure shows the putative 3-D structure of KIF3A and KIF3B. They all contain three domains: the head domain, the stalk domain, and the tail domain. They are all marked in different colors. (C) This figure shows the model pattern of the heterodimer containing KIF3A and KIF3B.

The analysis of the secondary structure and the 3-D structure were processed by PROSITE (http://prosite.expasy.org/) and I-TASSER (http://zhanglab.ccmb.med.umich.edu/I-TASSER). The results of the 3-D structure from I-TASSER were dealt with Vector NTI10 and Mega 5. The secondary structures of KIF3A and KIF3B are predicted to have three domains: the N-terminal, the stalk domain, and the C-terminal. As the protein structure has close relationship with its function, we predicted that the globular part is the head domain which is also the most conservative domain compared to the stalk domain and the tail domain. The stalk domain is the long and narrow part and is less conservative than the head domain. The tail domain is the rest part which comprise of nearly all the non-similar amino acids that are required for transporting different cargoes. The 1–420 amino acids of KIF3A and the 1–360 amino acids of KIF3B constitute the putative conserved domain that can move along the microtubules. The 421–570 amino acids of KIF3A and the 361–500 amino acids of KIF3B form the stalk domain. The 571–687 amino acids of KIF3A and the 501–587 amino acids of KIF3B form the tail domain that can carry different cargoes (Fig. 7).

### The expression of *kif3a* and *kif3b* in different tissues

**Figure 8:**
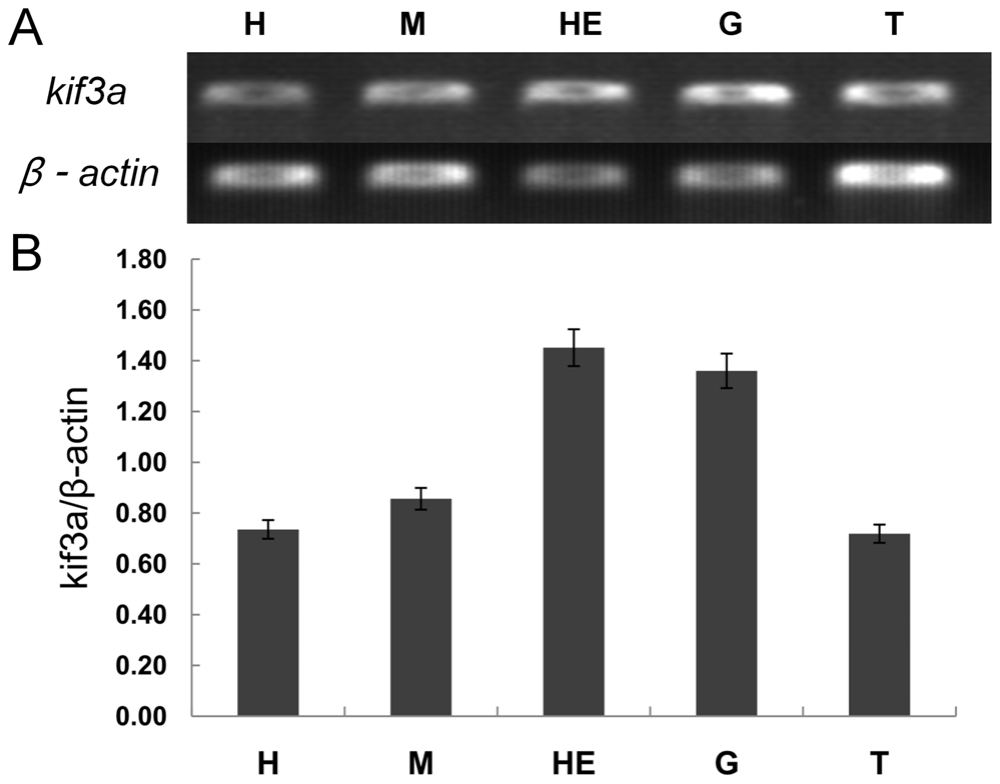
Semi-quantitative RT-PCR analysis of *kif3a* gene in different tissues. (A) This figure shows the expression of *kif3a* in different tissues of *E. sinensis* (upper panel). β-actin was used as a positive control (lower panel). The expression of *kif3a* is high in testis of *E. sinensis*. (B) This figure shows the quantitative analysis of the expression of *kif3a* in different tissues. *Kif3a* is highly expressed in the hepatopancreas and gill. The expression of *kif3a* in testis is the lowest of these tissues. H: heart, M: muscle, HE: hepatopancreas, G: gill, T: testis.

**Figure 9:**
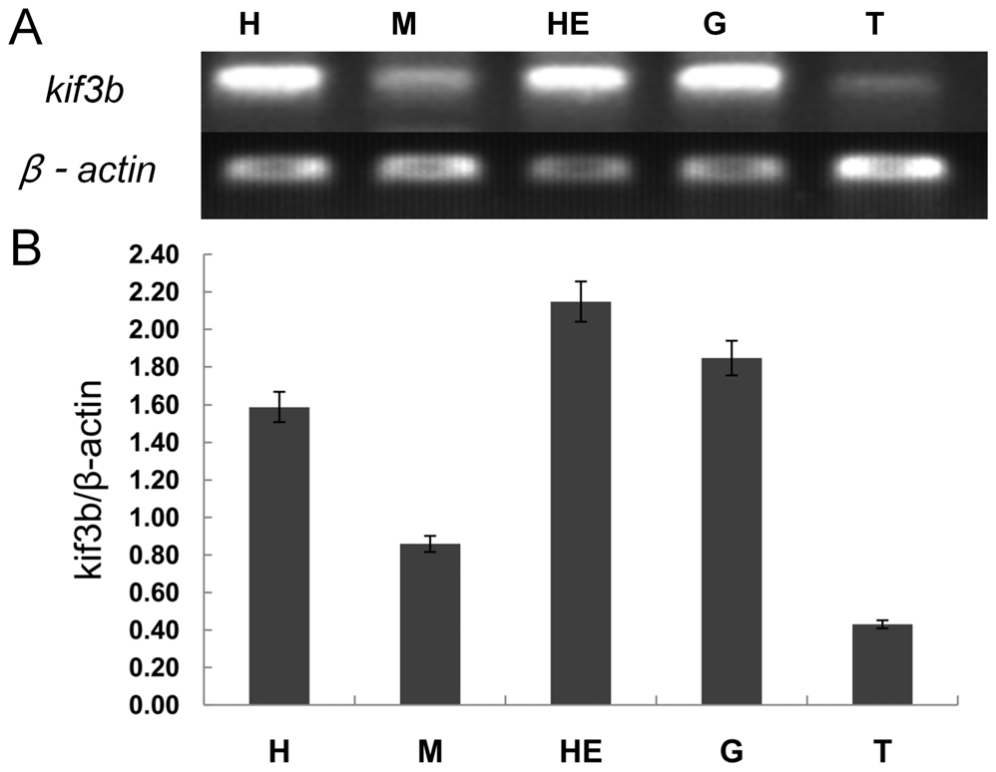
Semi-quantitative RT-PCR analysis of *kif3b* gene in different tissues. (A) This figure shows the expression of *kif3b* in different tissues of *E. sinensis* (upper panel). β-actin was used as a positive control (lower panel). The expression of *kif3b* is relatively low in testis of *E. sinensis*. (B) This figure shows the quantitative analysis of the expression of *kif3b* in different tissues. *Kif3b* is highly expressed in the hepatopancreas, gill, and heart. The expression of *kif3b* in testis is the lowest of these tissues. H: heart, M: muscle, HE: hepatopancreas, G: gill, T: testis.

We used the Semi-quantitative RT-PCR to analyze the expression of *kif3a* and *kif3b* in different tissues of *E. sinensis*. We amplified a 397 bp *kif3a* fragment and a 380 bp *kif3b* fragment from the heart, muscle, hepatopancreas, gill, and testis. *Kif3a* is highly expressed in the hepatopancreas and gill, while the expression of *kif3a* in testis is the lowest of these tissues (Fig. 8). *Kif3b* is highly expressed in the hepatopancreas, gill, and heart (Fig. 9). The expression of *kif3b* in testis is the lowest of these tissues.

### The temporal and spatial expression of *kif3a* and *kif3b* mRNA during spermiogenesis in *E. sinensis*

**Figure 10:**
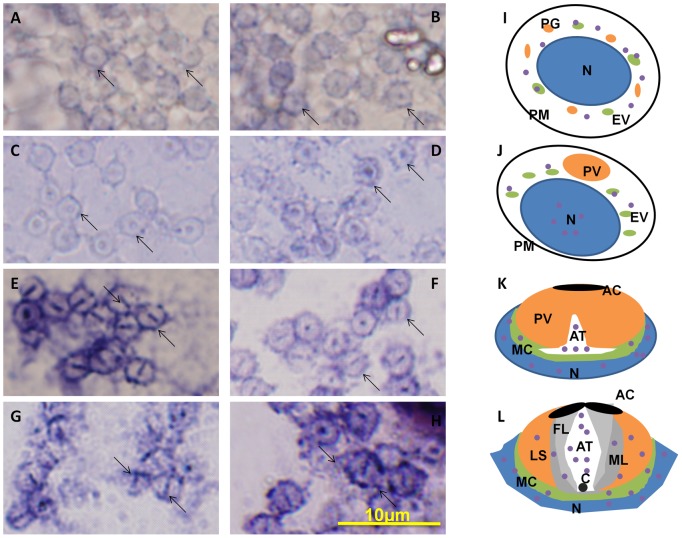
*In situ* hydridization of *kif3a* mRNA during spermiogenesis of *E. sinensis*. (A, B, I) Early stage of spermiogenesis. These figures show that *kif3a* mRNA signals (arrows; blue signal; purple dots) are weakly distributed in the cytoplasm of the round spermatids. (C, D, J) Middle stage of spermiogenesis. These figures show that *kif3a* mRNA signals (arrows; blue signal; purple dots) are not only distributed in the cytoplasm, but also concentrated on some parts of the nucleus. The expression of *kif3a* mRNA in the middle stage is larger than that in the early stage. (E, F, K) Late stage of spermiogenesis. These figures show that *kif3a* mRNA signals (arrows; blue signal; purple dots) are strongly distributed in the nucleus, the cytoplasm complex, the acrosomal tubule (AT) and the apical cap (AC). (G, H, L) Mature sperm. These figures show that *kif3a* mRNA signals (arrows; blue signal; purple dots) are mostly distributed in the acrosomal tubule (AT), the apical cap (AC), the cytoplasm complex and the nucleus. The three layers (fibrous layer FL, middle layer ML and lamellar structures LS) also have some weak signals of *kif3a* mRNA in these figures. The expression of *kif3a* was not decreased in this stage. (M) Control without mRNA. C: centriole.

**Figure 11:**
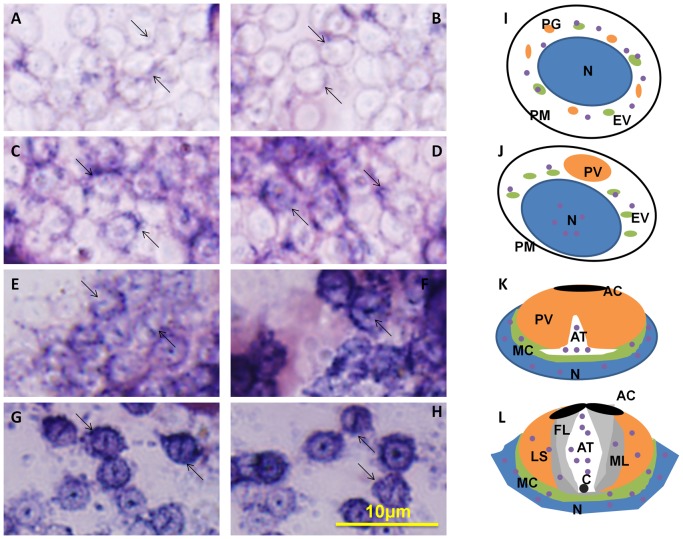
*In situ* hydridization of kif3b mRNA during spermiogenesis of *E. sinensis*. (A, B, I) Early stage of spermiogenesis. These figures show that *kif3b* mRNA signals (arrows; blue signal; purple dots) are weakly distributed in the cytoplasm of the round spermatids. (C, D, J) Middle stage of spermiogenesis. These figures show that *kif3b* mRNA signals (arrows; blue signal; purple dots) are distributed in the cytoplasm and some parts of the nucleus. The expression of *kif3b* mRNA in the middle stage is much higher than that in the early stage. (E, F, K) Late stage of spermiogenesis. These figures show that *kif3b* mRNA signals (arrows; blue signal; purple dots) are strongly distributed in the nucleus, the cytoplasm complex, the acrosomal tubule (AT) and the apical cap (AC). The signals of *kif3b* expression dramatically increased compared with the middle stage. (G, H, L) Mature sperm. These figures show that *kif3b* mRNA signals (arrows; blue signal; purple dots) are distributed in the acrosomal tubule (AT), the apical cap (AC), the cytoplasm complex, the nucleus, and the three layers (fibrous layer FL, middle layer ML and lamellar structures LS). The expression of *kif3b* was not decreased in this stage. (M) Control without mRNA. C: centriole.

ISH can be used to track the temporal and spatial expression of *kif3a* and *kif3b* mRNA. The localization of *kif3a* and *kif3b* relates to the biogenesis of the acrosome, and to the reshaping of nucleus (Fig. 10 and Fig. 11). At the early stage during spermiogenesis, *kif3a* and *kif3b* mRNA signals are weakly distributed in the cytoplasm of the round spermatids. They may have a similar localization and the same functions at the early stage (Fig. 10A, B and Fig. 11A, B, arrows; blue signal). Midway during spermiogenesis, the expression of *kif3a* mRNA signals remains almost the same, while the expression of *kif3b* mRNA signals shows some increase. The mRNA signals are not only distributed in the cytoplasm, but also concentrated in some parts of the nucleus (Fig. 10C, D and Fig. 11C, D, arrows; blue signal). At the late stage during spermiogenesis, the acrosomal tubule (AT) begins to form and the cytoplasm is concentrated as the cytoplasm complex (MC). The mRNA signals of *kif3a* and *kif3b* are primarily distributed in the nucleus, the cytoplasm complex, the acrosomal tubule (AT) and the apical cap (AC). The expression of the signals increase dramatically compared to the middle stage (Fig. 10E, F and Fig. 11E, F, arrows; blue signal). In the mature sperm, *kif3a* and *kif3b* mRNA signals (arrows; blue signal; purple dots) are mostly present in the acrosomal tubule (AT), the apical cap (AC), the cytoplasm complex and the nucleus. The three layers (fibrous layer FL, middle layer ML and lamellar structures LS) also have some weak signals (Fig. 10G, H and Fig. 11G, H, arrows; blue signal). A series of schematic patterns describe the temporal and spatial *kif3a* and *kif3b* mRNA during spermiogenesis (Fig. 10I, J, K and Fig. 11I, J, K). The controls (Fig. 10M and Fig. 11M) without adding riboprobes are shown to determine the staining background and to better identify the *kif3a* and *kif3b* signals.

### The expression of KIF3A and KIF3B in different tissues of *E. sinensis*

**Figure 12:**
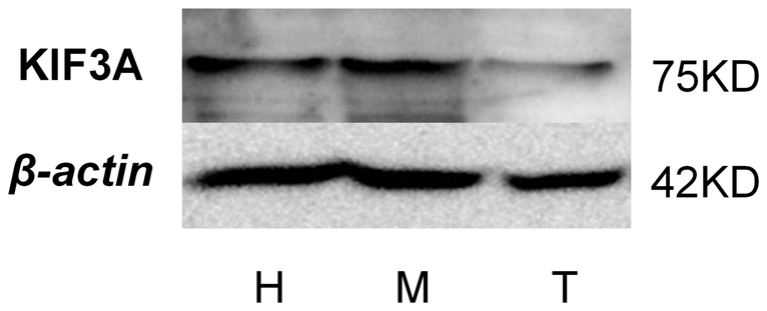
Western blot analysis of KIF3A in *E. sinensis*. The extracts of *E. sinensis* were probed with anti-KIF3A polyclonal antibody (upper panel). Anti-*β-actin* polyclonal antibody (lower panel) were also used to probe the tissue extracts). This figure shows that the expression of KIF3A protein is higher in the heart (H) and muscle (M) than it is in the testis (T). The molecular weight of *β-actin* is 42 KD and the molecular weight of KIF3A is about 75 KD.

The expression of KIF3A protein in *E. sinensis* heart, muscle, and testis is detected by Western blot. The KIF3A protein was detected as a possible 75KD band by anti-KIF3A polyclonal antibody (Fig. 12, upper panel). The *β-actin* was detected as a 42 KD band by anti-*β-actin* polyclonal antibody (Fig. 12, lower panel). From here, we can observe that the expression of KIF3A protein is higher in the heart (H) and muscle (M) than that in the testis (T).

### The temporal and spatial expression of KIF3A and KIF3B during spermiogenesis

**Figure 13:**
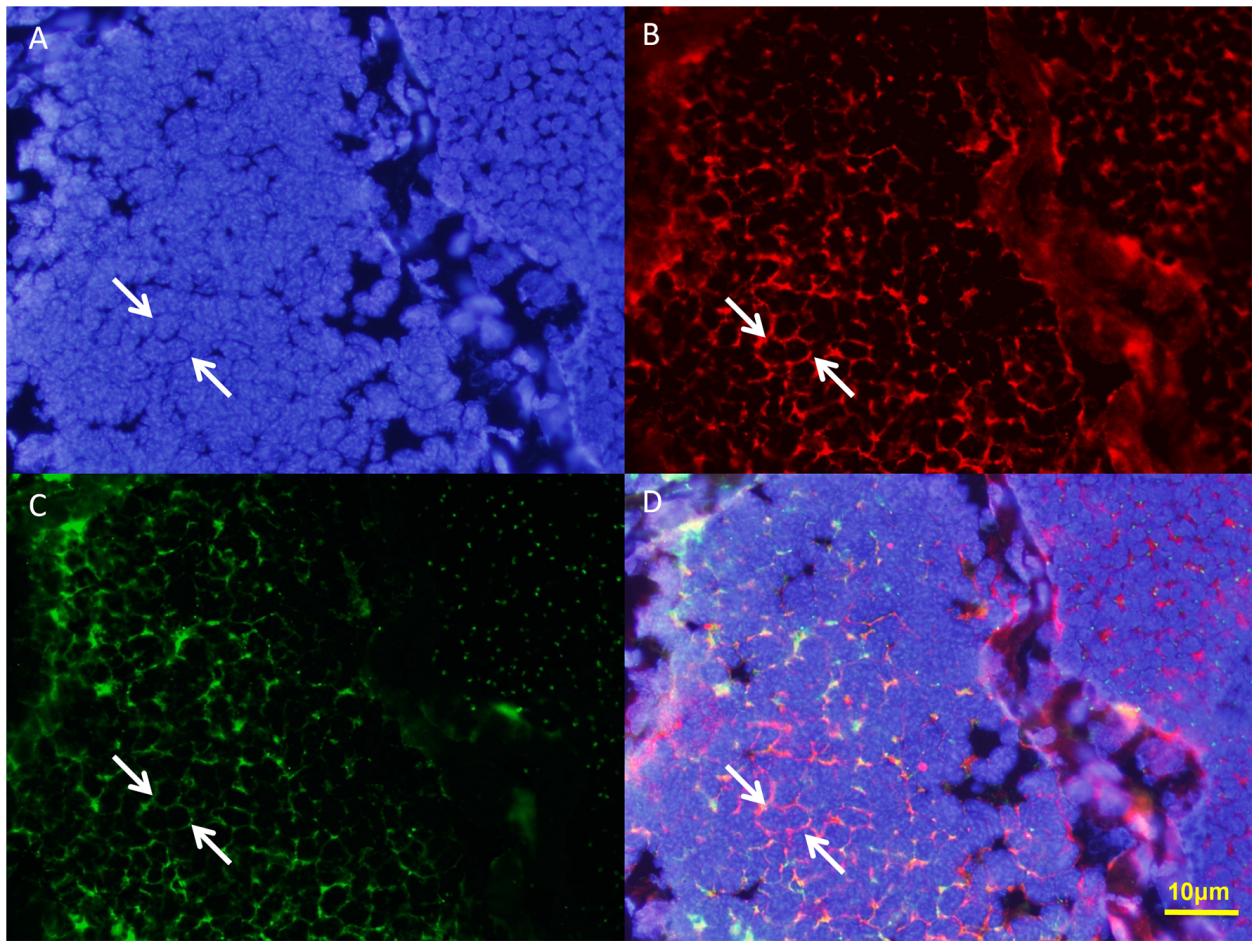
Immunofluorescent localization of KIF3A and tubulin in the early stage during spermiogenesis in *E. sinensis*. (A) DAPI nuclear staining (blue staining). (B) KIF3A staining (red staining). (C) Tubulin staining (green staining). This figure shows tubulin is localized mostly in the cytoplasm and a small part near the nuclear membrane. (D) Merged Immunofluorescent image. This figure shows that KIF3A and tubulin was co-localized in the cytoplasm in the early stage during spermiogenesis in *E. sinensis*. Nucleus (blue staining).

**Figure 14:**
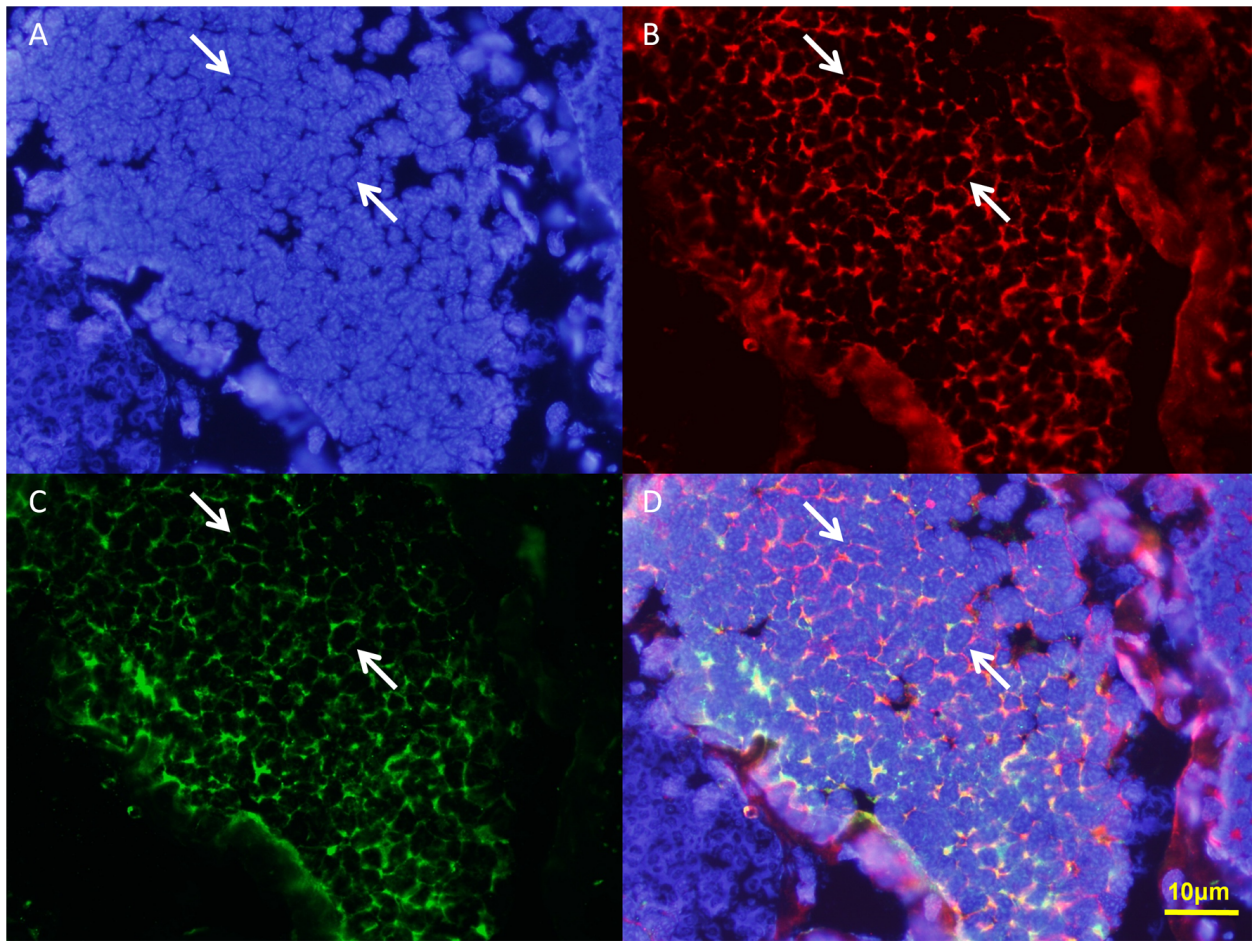
Immunofluorescent localization of KIF3A and tubulin in the middle stage during spermiogenesis in *E. sinensis*. (A) DAPI nuclear staining (blue staining). (B) KIF3A staining (red staining). (C) Tubulin staining (green staining). This figure shows that tubulin is localized mostly in the cytoplasm and a small part near the nuclear membrane. (D) Merged Immunofluorescent image. This figure shows KIF3A and tubulin was co-localized in the cytoplasm in the middle stage during spermiogenesis in *E. sinensis*. Nucleus (blue staining).

**Figure 15:**
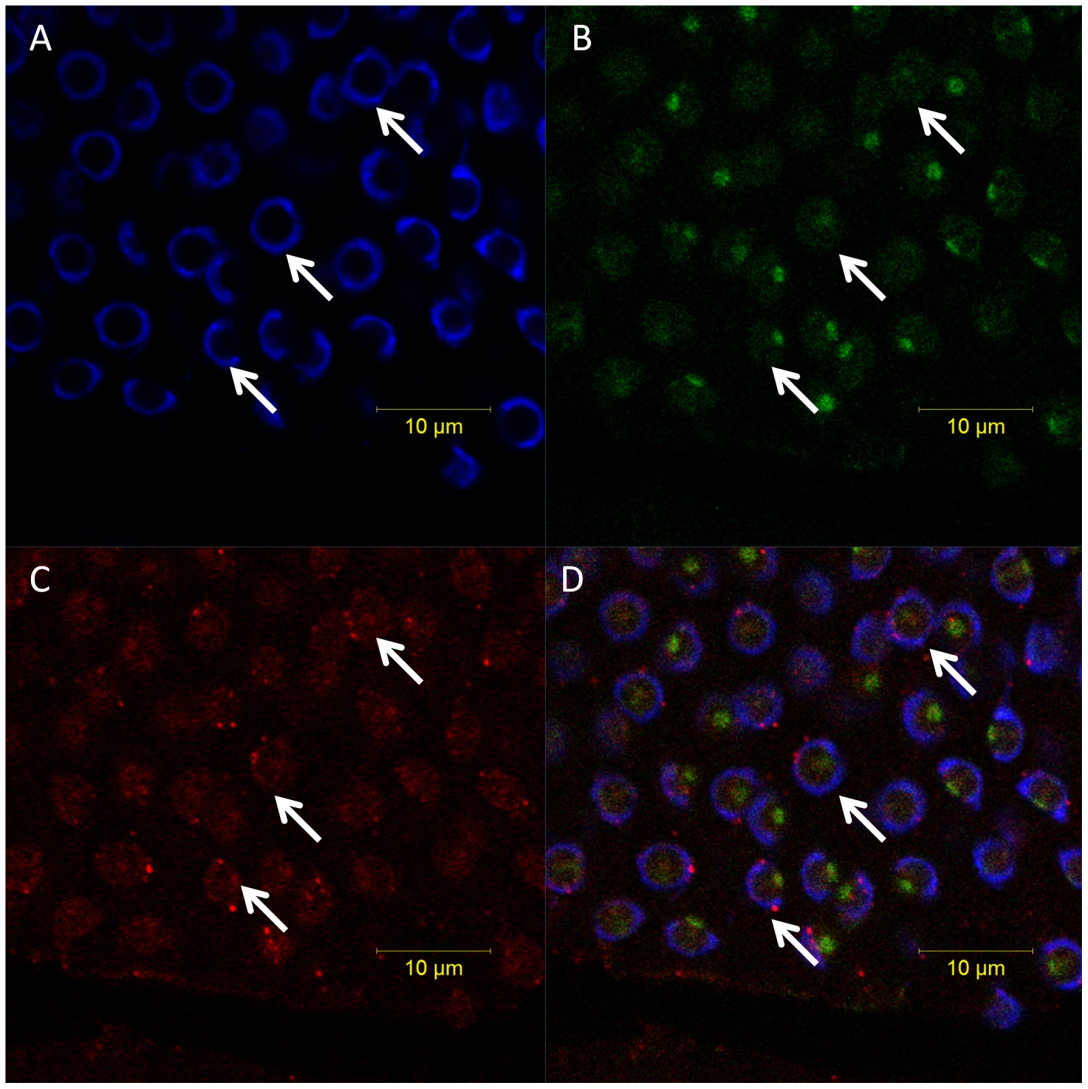
Immunofluorescent localization of KIF3A and tubulin in the mature sperm of *E. sinensis*. (A) DAPI nuclear staining (blue staining). (B) KIF3A staining (red staining). (C) Tubulin staining (green staining). This figure shows tubulin is localized in the cytoplasm complex, the acrosomal tubule and near the nuclear membrane. (D) Merged Immunofluorescent image. Tubulin (green staining; arrows) and KIF3A (red staining; arrows) localization is shown. Nucleus (blue staining).

Immunofluorescence (IF) is used to observe the localization of KIF3A in cellular transformations during spermiogenesis in *E. sinensis* (Fig. 13, 14, 15). KIF3A (Fig. 13B and D, arrows) signals are distributed in the cytoplasm of the round spermatids. KIF3A (Fig. 13B and D, arrows) and tubulin (Fig. 13C and D, arrows) is co-localized in the cytoplasm in the early stage during spermiogenesis in *E. sinensis*. At the middle stage during spermiogenesis, the proacrosomal granules develop into the proacrosomal vesicle; the nucleus begins to wrap the proacrosomal vesicle and becomes elongated. KIF3A (Fig. 14B and D, arrows) distributes in the cytoplasm of the elongate sperm. KIF3A (Fig. 14B and D, arrows) and tubulin (Fig. 14C and D, arrows) co-localize in the cytoplasm at the middle stage of spermiogenesis in *E. sinensis*. At the late stage of spermiogenesis, KIF3A (Fig. 15B and D, arrows) distributes in the acrosomal tubule (AT), the apical cap (AC), the cytoplasm complex and the nucleus. The three layers (fibrous layer, middle layer, and lamellar structures) also show some weak signals of *kif3b* mRNA.

## Discussion

In sexual reproduction, spermatogenesis is a vital developmental process providing a series of cellular transformations, such as the acrosome biogenesis, and the reshaping of nucleus and cytoplasm [42]. The morphology of the nucleus varies strikingly in different species. This indicates that morphological variability of the nucleus is related to the evolutionary divergence of taxa and sperm evolution. Almost all mammals and birds have a similar morphological process concerning the concentration and elongation of the nucleus, acrosome biogenesis, and the formation of a flagellar tail which is responsible for the movement of the sperm. During spermatogenesis, molecular motors can support a track for the transport and localization of components inside the sperm [42]. The kinesin superfamily proteins (KIFs) are responsible for intracellular transport of vesicles, membranous organelles, mRNA, and proteins in the neuron cells, somatic cells, and germ cells [44], [45]. KRP3A was reported to localize in the acrosome of the round spermatids and KRP3B was observed in the acrosome and on the surface of the nuclei [47]. KIF17 is reported to interact with Spatial-ε (an isoform of spatial, stromal protein associated with the thymus and lymph-node). So, spatial is the cargo of KIF17 in IFT and IMT with its specific role [48], [49]. KIF17 is also responsible for the transport of RNA and transcription mediators shuttling between nucleus and cytoplasm, such as ACT the activator of CREM [43], [48], [49], [50]. As for KIF3A and KIF3B, they are speculated to function in the manchette, a special microtubule-based structure, and the flagellar tail during spermatogenesis, [3], [4], [5], [36], [46]. A series of transformations related to the mobilization and the reorganization of the cytoskeleton network, the transport of intracellular organelles and complexes, and the reshaping of some organelles have relations to KIF3A and KIF3B [3], [4], [5]. However, due to the peculiar shape of the spermatozoon in *E. sinensis* compared to other species, it may also have particular functions and mechanisms during spermatogenesis. Apparently, the sperm of *E. sinensis* has no flagellar tail, but has a peculiar process of acrosome development [1], [7]. Whether KIF3A and KIF3B are responsible for the cellular transformation during spermiogenesis of *E. sinensis* needs further investigation.

In the present study we cloned *kif3a* and *kif3b* from the testis of *E. sinensis*. The phylogenetic tree and multiple sequence alignment between *E. sinensis* and other species illustrates that KIF3A and KIF3B are relatively conserved in this species during evolution. They have the conservative domains containing microtubule-binding domains and ATP-binding domains. The prediction of the structures of KIF3A and KIF3B reflect that they all have an N-terminal domain constructing the head, the coiled-coil stalk, and the C-terminal domain constructing the tail (Fig. 7). As KIF3A and KIF3B can form a heterodimer at the coiled-coil stalk in many species, we speculate that KIF3A and KIF3B of *E. sinensis* can also form a heterodimeric complex based on the interaction of the stalk region [3], [4], [5]. The N-terminal region can bind to the microtubule for walking along, while the C-terminal region can recognize different cargoes. They may have a vital role in the evolution of different species. Although they represent a low expression in testis compared to other tissues, such as heart, muscle, and gill in *E. sinensis*, they also have a vital function during spermatogenesis. But considering the strong expression in the testis of other species, such as *Cynops orientalis* and *Octopus tankahkeei*, they may have a different expression pattern and special functions in the testis of *E. sinensis*. *In situ* hydridization (ISH) reveals the expression distribution of *kif3a* and *kif3b* mRNA and Immunofluorescence (IF) reveals the expression distribution of KIF3A protein. At the early stage during spermiogenesis, *kif3a* and *kif3b* mRNA signals are weakly distributed in the cytoplasm of round spermatids. At the middle stage, the mRNA signals are not only distributed in the cytoplasm, but are also concentrated in some parts of the nucleus. At a later stage, the mRNA signals of *kif3a* and *kif3b* are strongly distributed in the nucleus, the cytoplasm complex, the acrosomal tubule (AT) and the apical cap (AC) (Fig. 10, 11). In the mature sperm, *kif3a* and *kif3b* mRNA signals are mostly distributed in the acrosomal tubule (AT), the apical cap (AC), the cytoplasm complex and the nucleus. The three layers (fibrous layer FL, middle layer ML and lamellar structures LS) also have some weak signals (Fig. 10, 11). The KIF3A protein shows the same distribution (Fig. 15).

At the early stage of spermatogenesis, the finding that they mainly localize in the cytoplasm near the nucleus reveals that KIF3A and KIF3B as microtubule-dependent motor proteins can walk along the cytoskeleton of the sperm. The mechanism of PV formation is believed to interact closely with the ER, but there is evidence that the Golgi apparatus is also responsible for the formation of PV [14]. At the middle stage, there are also weak expressions of KIF3A and KIF3B concentrating on the nucleus. KIF17 was reported to be responsible for the transport of RNA and transcription mediators shuttling between nucleus and cytoplasm such as ACT, activator of CREM [43], [48], [49], [50]. CREM, cAMP-responsive element modulator, plays a key role in the maturation and differentiation of spermatids. Whether KIF3A and KIF3B also have the same function in transporting similar factors need further investigation. At the late stage of spermatogenesis, the AT begins to form and the nucleus becomes sharply cup-shaped. KIF3A and KIF3B strongly distributes in the nucleus, the cytoplasm complex, the acrosomal tubule (AT) and the apical cap (AC)(Fig. 11). In the mature sperm, these proteins are mostly distributed in the acrosomal tubule (AT), the apical cap (AC), the cytoplasm complex and the nucleus. The three layers (fibrous layer, middle layer and lamellar structures) also have some weak signals. The finding here is different from the distribution of KIF3A and KIF3B in the testis of other species [3], [4], [5]. Several proteins have vital roles in the formation of the acrosome, KIF3A and KIF3B may function in reshaping of the nucleus, the biogenesis of AT and the maintenance of the acrosome through transporting cargoes. Later penetration of sperm depends on the perforatorium functions. KIF3A and KIF3B may have some roles in the acrosome during fertilization [51] since the subacrosomal space is filled with actin instead of microtubules [52]. However, kinesin and myosin were reported to function together in the cytoskeleton and promote mutual functions to facilitate motility [53], [54], [55]. In addition, KIFC1 was suggested to bind to actin with the assistance of myosin [14]. So, we suggest that KIF3A and KIF3B may also have a similar role during spermatogenesis. However, the exact function and its mechanism of KIF3A and KIF3B in the acrosome remain to be studied further. The same holds for the cargoes of KIF3A and KIF3B in the course of spermatogenesis.

In conclusion, KIF3A and KIF3B can assemble as a heterodimer to walk along the microtubule. KIF3A and KIF3B play roles in the biogenesis of the acrosome, the reshaping of nucleus and cytoplasm, and fertilization by transporting different cargoes and vesicles.
